# Supplementary material for: Hypermetabolic state is associated with circadian rhythm disruption in mouse and human cancer cells
Source: Proc Natl Acad Sci U S A. 2024 Jul 15;121(30):e2319782121. doi: 10.1073/pnas.2319782121 (PMC11287162; doi:10.1073/pnas.2319782121)
Supplement: Supplementary file 1 — Appendix 01 (PDF) [file pnas.2319782121.sapp.pdf]

PNAS

Supporting Information for
Hypermetabolic state is associated with circadian rhythm disruption in mouse and human cancer cells

Daniel Maxim Iascone[1], Xue Zhang[2,3,8], Patricia Brafford[2,3], Clementina Mesaros[4], Yogev Sela[2,5], Samuel Hofbauer[4], Shirley L. Zhang[1,9], Sukanya Madhwal[1], Kieona Cook[6,7], Pavel Pivarshev[1], Ben Z. Stanger[2,5], Stewart Anderson[6,7], Chi V. Dang[2,3,8], Amita Sehgal[1]

1. Howard Hughes Medical Institute, Chronobiology and Sleep Institute, Perelman School of Medicine, University of Pennsylvania, Philadelphia, PA, USA
2. Abramson Cancer Center, University of Pennsylvania, Philadelphia, PA 19104
3. Wistar Institute, Philadelphia, PA, USA
4. Department of Systems Pharmacology and Translational Therapeutics, University of Pennsylvania, Philadelphia, PA, USA
5. Department of Cell and Developmental Biology, Perelman School of Medicine at the University of Pennsylvania, Philadelphia, Pennsylvania, USA
6. Department of Psychiatry, Perelman School of Medicine, University of Pennsylvania, Philadelphia, Pennsylvania, USA
7. Department of Child and Adolescent Psychiatry, Children's Hospital of Philadelphia, Philadelphia, Pennsylvania, USA
8. Present address: Johns Hopkins University, Baltimore, MD, USA
9. Present address: Emory University, Atlanta, GA, USA

Corresponding Author
Amita Sehgal
Email: amita@pennmedicine.upenn.edu

This PDF file includes:

Figures S1 to S8
Tables S1 to S2

Other supporting materials for this manuscript include the following:

Datasets S1 to S2

Supplementary Figure 1

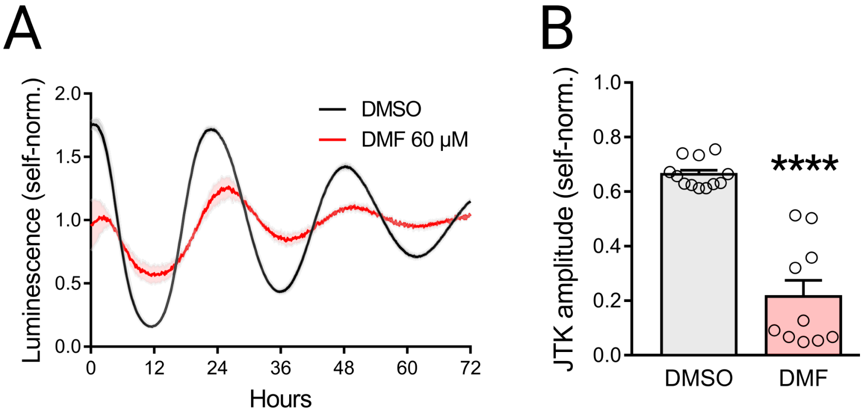

Supplementary Figure 1. Nrf2 agonist DMF suppresses circadian rhythms in MAFs (**A**) Self-normalized luminescence traces recorded from Bmal1::luciferase MAFs treated with DMF (n = 10-12 replicates from 3 experiments). (**B**) Oscillation amplitudes of traces from (A). Error bars indicate mean ± SEM. ****$p < 0.0001$, Student's t-test.

Supplementary Figure 2

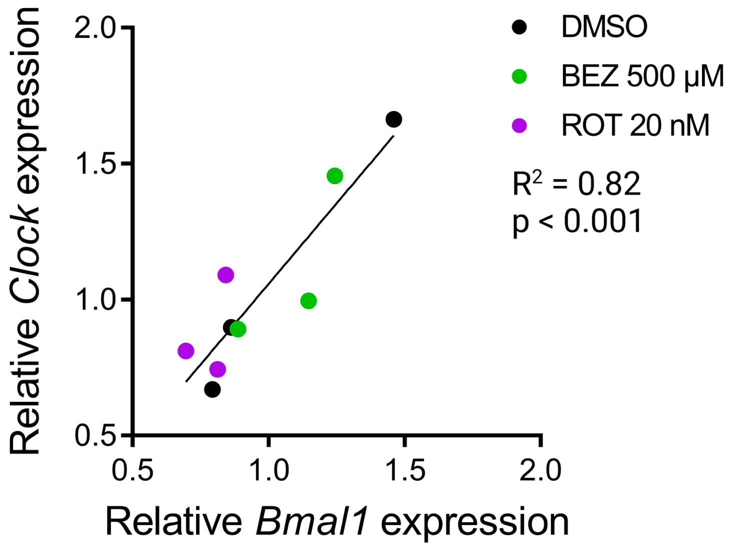

Supplementary Figure 2. Association between *Bmal1* and *Clock* in MAFs Linear correlation between peak *Bmal1* and *Clock* mRNA expression across 48-hour drug treatment groups measured with qPCR relative to *Actin*. Each point represents an average of 3 technical replicates. R-squared and p values were calculated using a simple linear regression model.

Supplementary Figure 3

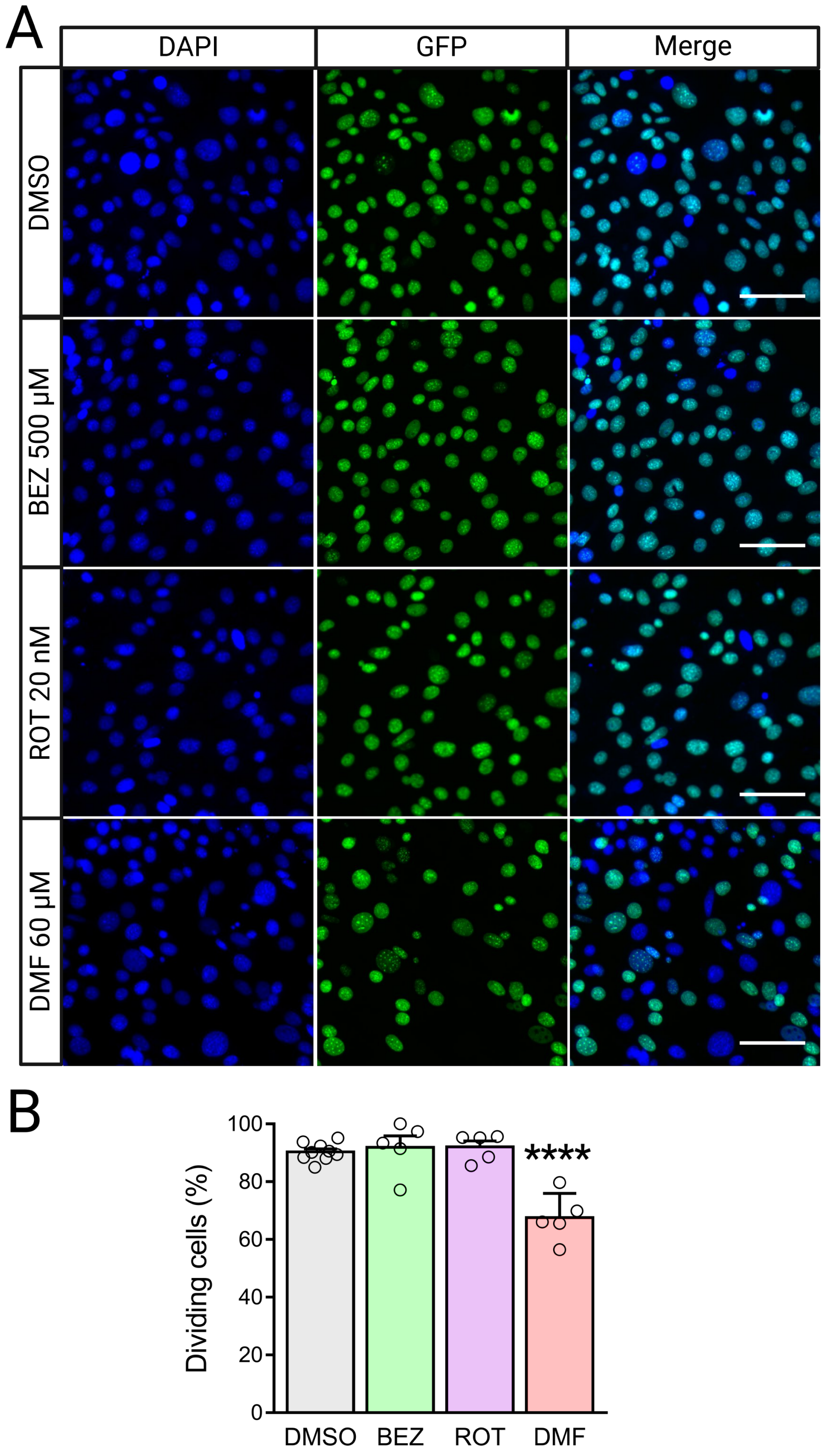

Supplementary Figure 3. MAF cell division following drug treatment (**A**) Representative images of DAPI and EdU nuclei staining in MAF cultures following 48 hours of treatment with BEZ, ROT, or DMF (**B**) Percentage of EdU^+ nuclei (dividing cells) from MAF cultures following drug treatment (n = 6-9 ROIs from 2-3 biological replicates). Scale bars: 100 μm. Error bars indicate mean ± SEM. ****$p < 0.0001$, one-way ANOVA test with Benjamini, Krieger, and Yekutieli's two-stage step-up procedure to control the FDR, drug treatment conditions compared to DMSO control.

Supplementary Figure 4

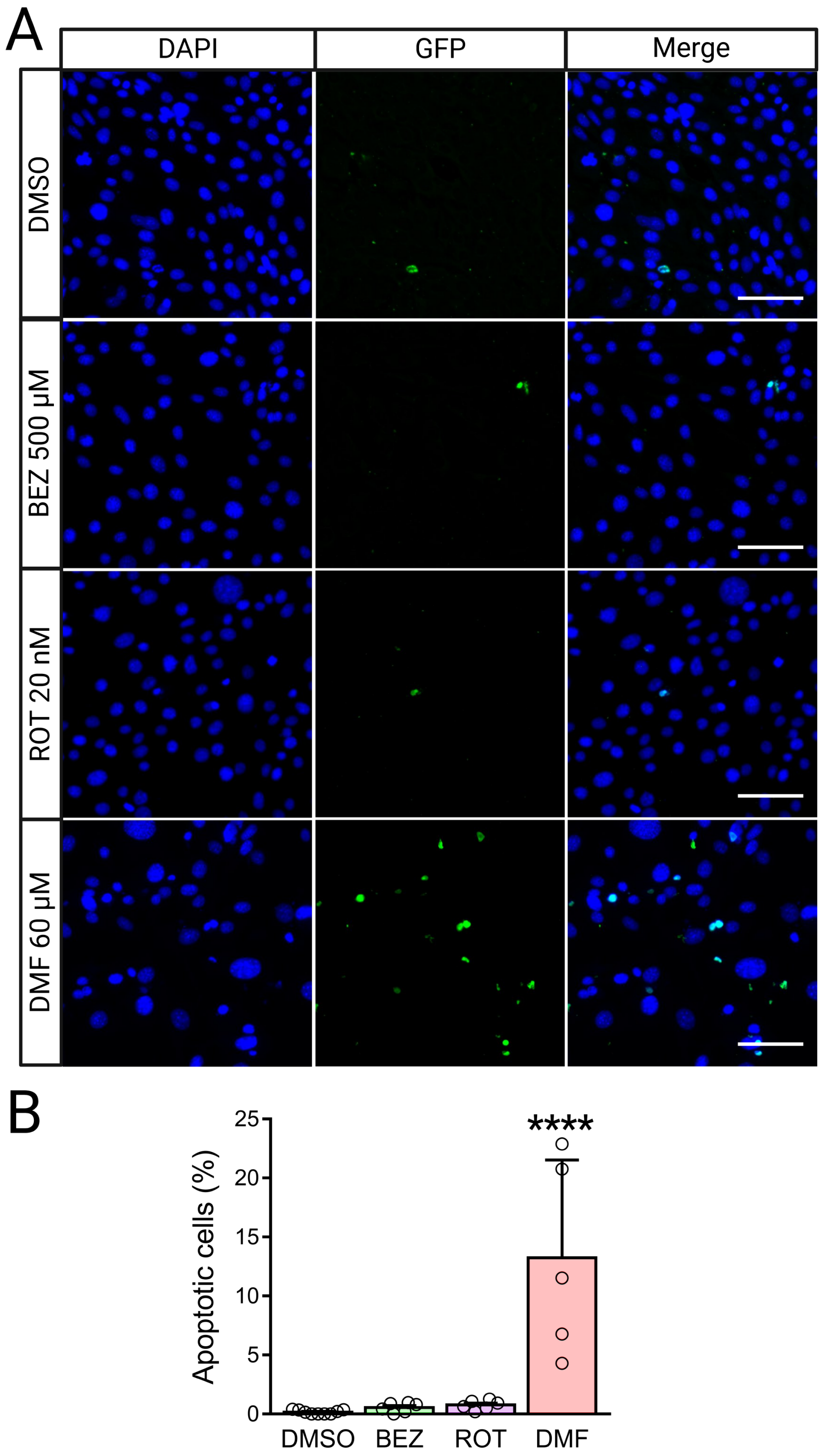

Supplementary Figure 4. MAF apoptosis following drug treatment (**A**) Representative images of DAPI and TUNEL staining in MAF cultures following 48 hours of treatment with BEZ, ROT, or DMF (**B**) Percentage of TUNEL^{+} particles (apoptotic cells) from MAF cultures following drug treatment (n = 6-9 ROIs from 2-3 biological replicates). Scale bars: 100 μm. Error bars indicate mean ± SEM. ****$p < 0.0001$, one-way ANOVA test with Benjamini, Krieger, and Yekutieli's two-stage step-up procedure to control the FDR, drug treatment conditions compared to DMSO control.

Supplementary Figure 5

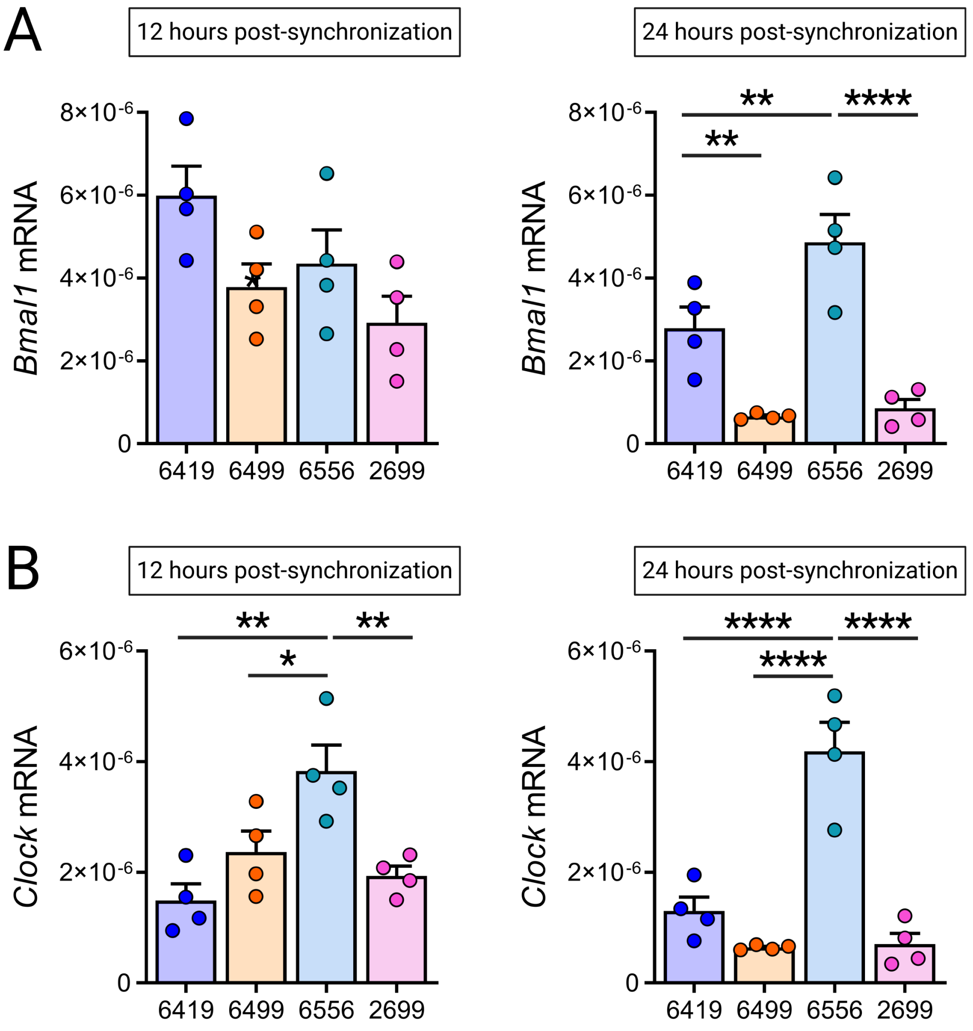

Supplementary Figure 5. *Bmal1* and *Clock* expression across PDA cell lines (**A-B**) qPCR measurement of *Bmal1* (**A**) and *Clock* (**B**) expression relative to *Rplp0* of PDA cell lines 12 and 24 hours following dexamethasone synchronization (n = 4 biological replicates; each point represents an average of 3 technical replicates). Error bars indicate mean ± SEM. *p < 0.05, **p < 0.01, ****p < 0.0001, one-way ANOVA test with Benjamini, Krieger, and Yekutieli's two-stage step-up procedure to control the FDR.

Supplementary Figure 6

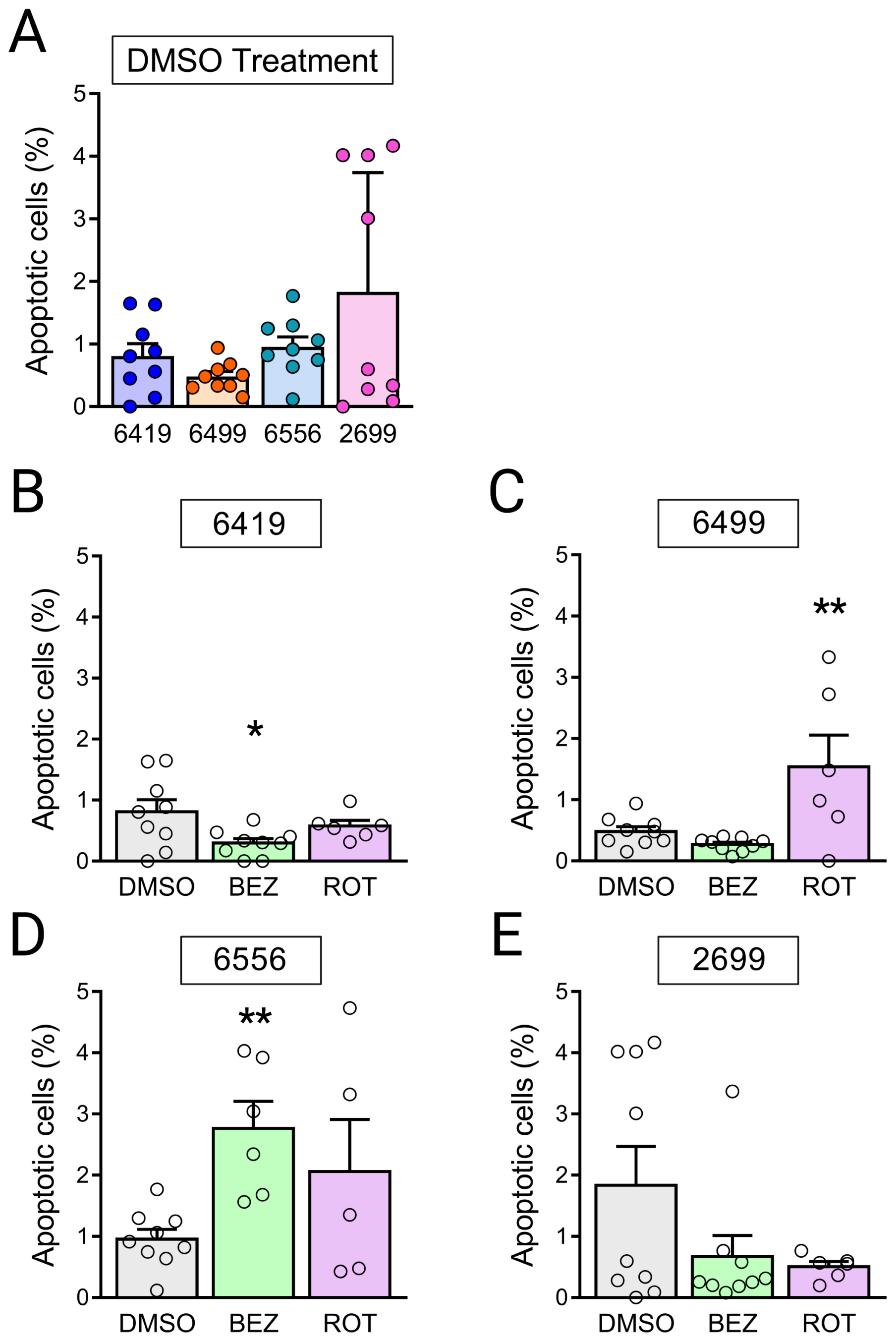

Supplementary Figure 6. PDA apoptosis following metabolic drug treatment (**A**) Percentage of TUNEL$^+$ cells from PDA cell lines following DMSO treatment (n = 9 ROIs from 3 biological replicates). (**B-E**) Percentage of TUNEL$^+$ cells from PDA cell lines 6419 (D), 6499 (C), 6556 (D), and 2699 (E) following metabolic drug treatment (n = 6-9 ROIs from 2-3 biological replicates). Error bars indicate mean ± SEM. *p < 0.05, **p < 0.01, one-way ANOVA test with Benjamini, Krieger, and Yekutieli's two-stage step-up procedure to control the FDR, drug treatment conditions compared to DMSO control for (B-E).

Supplementary Figure 7

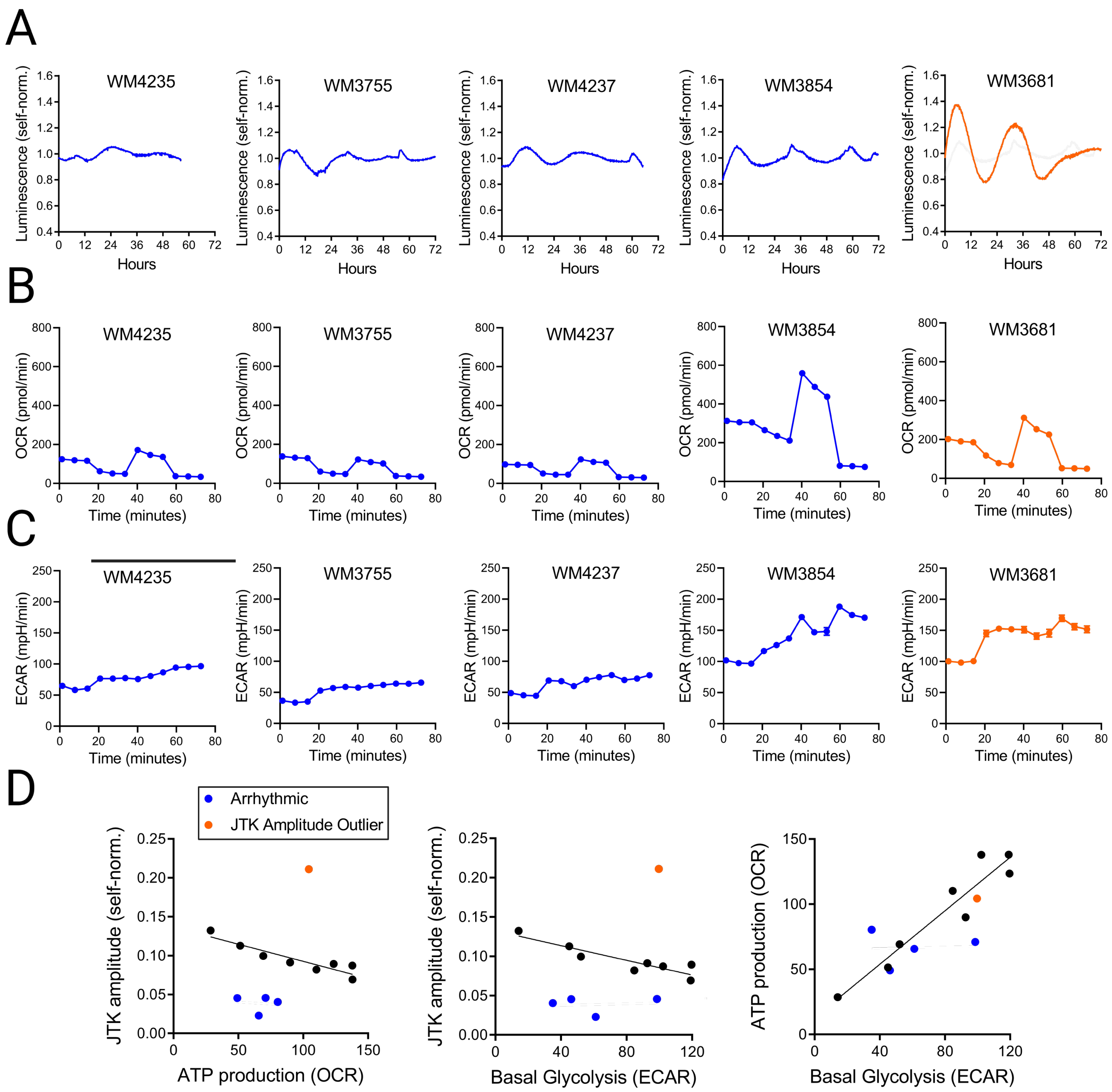

Supplementary Figure 7. Circadian and metabolic phenotypes of excluded patient-derived melanoma lines (**A**) Self-normalized luminescence traces ± SEM of Bmal1::luciferase activity recorded from melanoma cell lines excluded due to arrhythmicity (blue) or outlier cycling amplitude (orange; n = 2 biological replicates). (**B**) Oxygen consumption rates of melanoma cell lines during Seahorse mitochondrial stress test. (**C**) Extracellular acidification rates of melanoma cell lines during Seahorse mitochondrial stress test. (**D**) Excluded melanoma lines plotted relative to linear correlations between JTK amplitude and ATP production (left), JTK amplitude and basal glycolysis (middle), and ATP production and basal glycolysis (right) across melanoma cell lines. Error bars indicate mean ± SEM.

Supplementary Figure 8

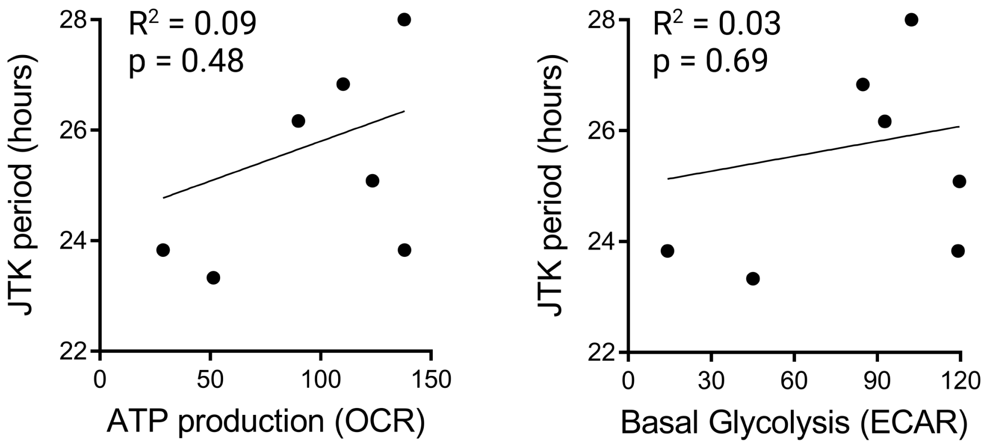

Supplementary Figure 8. No association between circadian period and metabolic phenotypes across patient-derived melanoma cell lines Linear correlations between JTK period (hours) and either ATP production (left) or basal glycolysis (right) across melanoma cell lines. R-squared and p values were calculated using a simple linear regression model.

Supplementary Table 1

Internal Standards (ISTD) (labeled compounds) added to each plate before metabolomic extraction

Internal Standard (ISTD)	Concentration in Mix (ng μL^{-1})	Added Amount (ng per plate)	Supplier
**Metabolomics ISTD Mix**			
Glycine – [13]C2, [15]N	11	440	CIL
Serine – [13]C3, [15]N	60	2400	CIL
Isoleucine – [13]C6, [15]N	10.5	420	CIL
Thymidine – [15]N2	11.5	460	CIL
Threonine – [13]C4, [15]N	11	440	SA
Pyruvate – [13]C3	10	400	CIL
Arginine – [13]C6	9.5	380	CIL
Valine – [13]C5, [15]N	110	4400	CIL
Tryptophan – [13]C11	11.5	460	CIL
Lysine – [13]C6	10	400	CIL
Aspartic Acid – [13]C4, [15]N	10.5	420	CIL
Glutamic Acid – [13]C5, [15]N	10	400	SA
Glutamine – [13]C5, [15]N	10	400	SA
Gluconolactone – [13]C6	50	2000	TRC
D-3-Hydroxybutyrate – [13]C4	10.5	420	CIL
Adenosine – [13]C5	50	2000	CIL
Ketoglutarate – D5	2.5	100	CIL
Hydroxyglutarate – [13]C5	2.5	100	SA
Oxalic Acid – [13]C2	5	200	TRC
Fumaric Acid – [13]C4	N/A	N/A	CIL
Succinic Acid – [13]C4	11	440	SA
Malonic Acid – [13]C3	0.5	20	TRC
Fructose 6-Phosphate – [13]C6	5	200	TRC
Citric Acid – [13]C6	9	360	SA
Fructose 1,6-Bisphosphate – [13]C6	12.5	500	TRC
Acetyl-CoA – [13]C2	2.5	100	SA

CIL = Cambridge Isotope Laboratories, Inc., SA = Sigma-Aldrich, TRC = Toronto Research Chemicals, Inc.

Supplementary Table 2

Experiment Plating Parameters

Figure	Assay Type	Cell Type	Plate Type	**Seeding Density** (cells per well)	**Pre-treatment Time** (hours before drug treatment prior to assay)	**Pre-assay Treatment Time** (hours of drug treatment before assay)	**Assay Treatment Time** (hours of drug treatment during assay)	Drug Treatments
1B-C	Seahorse Mito Stress Test	NIH3T3 MAF	96-well	15000	24	48	N/A	DMSO (control); 500 µM bezafibrate; 20 nM rotenone
1D	qPCR	NIH3T3 MAF	35 mm	1000000	24	48	N/A	DMSO (control); 500 µM bezafibrate; 20 nM rotenone
1E-F	Luciferase recording	NIH3T3 MAF	24-well	100000	24	24	84	DMSO (control); 500 µM bezafibrate; 20 nM rotenone
3	Luciferase recording	PDA	24-well	100000	24	N/A	N/A	N/A
4	Luciferase recording	PDA	24-well	100000	24	24	84	DMSO (control); 500 µM bezafibrate; 20 nM rotenone
5A	Luciferase recording	Wistar Melanoma (WM)	24-well	40000	24	N/A	N/A	N/A
5B-C	Seahorse Mito Stress Test	Wistar Melanoma (WM)	96-well	30000	24	N/A	N/A	N/A
S1	Luciferase recording	NIH3T3 MAF	24-well	100000	24	24	84	DMSO (control); 60 µM dimethyl fumarate
S3	EdU cell division assay	NIH3T3 MAF	96-well	10000	24	48	N/A	DMSO (control); 500 µM bezafibrate; 20 nM rotenone; 60 µM dimethyl fumarate
S4	TUNEL apoptosis assay	NIH3T3 MAF	96-well	10000	24	48	N/A	DMSO (control); 500 µM bezafibrate; 20 nM rotenone; 60 µM dimethyl fumarate
S5	qPCR	PDA	24-well	500000	18 or 30	N/A	N/A	N/A
S6	TUNEL apoptosis assay	PDA	96-well	10000	24	48	N/A	DMSO (control); 500 µM bezafibrate; 20 nM rotenone
S7A	Luciferase recording	Wistar Melanoma (WM)	24-well	40000	24	N/A	N/A	N/A
S7B-C	Seahorse Mito Stress Test	Wistar Melanoma (WM)	96-well	30000	24	N/A	N/A	N/A
